# Bacterial isolates from blood cultures of children with suspected septicaemia in Calabar, Nigeria

**DOI:** 10.1186/1471-2334-5-110

**Published:** 2005-12-08

**Authors:** Martin M Meremikwu, Chukwuemeka E Nwachukwu, Anne E Asuquo, Joseph U Okebe, Simon J Utsalo

**Affiliations:** 1 Departments of Paediatrics, Faculty of Clinical Sciences, University of Calabar, Calabar, Nigeria; 2 Department of Medical Microbiology and Parasitology, Faculty of Laboratory and Allied Health Sciences, University of Calabar, Calabar, Nigeria

## Abstract

**Background:**

Septicaemia is a common cause of morbidity and mortality among children in the developing world. This pattern has changed little in the past decade. Physical signs and symptoms, though useful in identifying possible cases have limited specificity. Definitive diagnosis is by bacteriologic culture of blood samples to identify organisms and establish antibiotic susceptibility. These results are usually not available promptly. Therefore a knowledge of epidemiologic and antimicribial susceptibility pattern of common pathogens is useful for prompt treatment of patients. This report highlights the pattern of bacterial isolates in our environment from a retrospective study of our patients' records.

**Methods:**

One thousand, two hundred and one blood samples were analysed from children aged 0–15 years, admitted into the children's wards of the University of Calabar Teaching Hospital, Calabar, Nigeria with features suggesting septicaemia. Samples were collected under aseptic conditions and cultured for aerobic and anaerobic organisms. Isolates were identified using bacteriologic and biochemical methods and antibiotic sensitivity determined by agar diffusion method using standard antibiotic discs.

**Results:**

Bacteria was isolated in 552 (48.9%) of samples with highest rates among newborns (271 : 50.8). The most frequent isolates were *Staphylococcal aureus *(48.7%) and *Coliforms *(23.4%). Results showed high susceptibilities to the Cephalosporins (Ceftriazone- 100%:83.2%, Cefuroxime-100%:76.5%) and Macrolides (Azithromycin-100%:92.9%) for *S. aureus *and *coliforms *respectively. This study underscores the importance of septicaemia as a common cause of febrile illness in children and provides information on common prevalent aetiologic agents and drug susceptibilities of the commonest pathogens.

**Conclusion:**

*Staphylococcus aureus *and *coliforms *were the leading causes of septicaemia in children in this locality, and the third generation cephalosporins and azithromycin were shown to be effective against these pathogens.

## Background

Septicaemia is a common cause of paediatric morbidity and mortality. Deaths from paediatric septicaemia are likely to be higher in low-income settings. Children with septicaemia present with fever, difficult breathing, tachycardia, malaise, inability to feed or lethargy, but those with asymptomatic bacteraemia tend to show no obvious sign of illness. In Nigeria, septicaemia is a major cause of death in neonates and children [1,2]. The outcome of treatment of neonates with septicaemia has remained poor in Nigeria as shown by reports of mortality rate of 33% to 41% from two tertiary hospitals in the country [3,4].

Prompt diagnosis and effective treatment is necessary to prevent death and complications from septicaemia. Physical signs and symptoms are useful in identifying infants and children with septicaemia and other non-localised infections but these have limited specificity [5,6]. Clinical assessment using a combination of symptoms and signs are useful guides to provisional diagnosis of septicaemia. For instance, a seven-item, weighted, clinical score system comprising grunting, abdominal distension, increased pre-feed aspirates, tachycardia, hyperthermia, chest retractions and lethargy showed that these criteria were sensitive for identifying newborns with septicaemia [7].

Rapid immunological techniques like C-Reactive Proteins (CRP) assays may help in the preliminary diagnostic assessment of suspected septicaemia. However, they lack the capacity to detect specific pathogens. Bacteriological culture to isolate the offending pathogen remains the mainstay of definitive diagnosis of septicaemia. The results of bacteriological cultures and antibiotic susceptibility tests take about a week, necessitating initial empirical treatment of suspected septicaemia. Knowledge of epidemiological and anti-microbial susceptibility pattern of common pathogens in a given area helps to inform the choice of antibiotics. We report the pattern of bacterial isolates in children with clinical diagnosis of septicaemia seen at the University of Calabar Teaching Hospital in South-eastern Nigeria.

## Methods

The study included all consecutive blood cultures in children aged 0–15 years admitted to the University of Calabar Teaching Hospital (UCTH) from July 1996 to December 2002. The indication for blood cultures were clinical features adjudged by the attending clinician to be indicative of sepsis especially fever without localized lesion and absence of asexual malaria parasite in peripheral blood. Presence of malaria parasitaemia did not preclude suspicion of septicaemia when illness was severe or risk factors of sepsis such as severe malnutrition, lethargy and abdominal distension were present.

Specimens were collected into cooked meat broth under aseptic conditions using sterile, disposable hypodermic needle and syringe and transported to the laboratory within 30 minutes of collection. Aerobic cultures were mounted on the same day and sub-cultured within 48 hours if there was any indication of growth. Eosin methylene blue and blood agar plates were used for sub-culture and incubated at 37°C aerobically and in candle extinction jar respectively. Full identification of organisms was done with standard bacteriological and biochemical methods [8]. Antibiotic sensitivity patterns of bacterial isolates were determined by agar diffusion method using antibiotic discs [9]

## Results

A total of 1,201 children with suspected septicaemia were studied. The age and sex distribution of the patients are shown in Table 1. There were 539 (44.9%) females and 662 (55.1%) males. The majority of the patients were newborns (533; 44.4%) and infants (252; 21.0%).

**Table 1:** Age and sex distribution of 1201 children with suspected septicaemia in Calabar Nigeria

Age	Number of Children Examined			Number (%) with positive bacterial isolates
		
	Female	Male	Total	
< 1 mo	230	303	533	271 (50.8)
1 mo – 1 yr	111	141	252	113 (44.8)
2 yr – 5 yr	126	133	259	113 (43.6)
6 yr – 10 yr	40	55	95	39 (41.0)
11 yr – 18 yr	32	30	62	16 (25.8)
Total	539	662	1201	552 (46.0)

Bacteria were isolated in 552 (45.9%) of the 1,201 patients studied. The types and pattern of bacterial isolates in age groups is shown on Table 2. The rate of isolation was highest among newborns (271/533: 50.8%). The overall rate of isolation reduced with increasing age but the types of organisms cultured did not vary with age. The most frequent isolates were *Staphylococcus aureus *(48.7%) and *Coliforms *(23.4%). Unidentified gram-negative rods (8.0%), *Pseudomonas aeruginosa spp *(5.8%), *Streptococcal spp *(4.7%) and *Chromobacterium spp *(4.5%) were also fairly frequently identified. We did not isolate anaerobes. Our laboratory techniques may not have been sensitive enough to detect obligate anaerobes.

**Table 2:** Age distribution of 552 bacterial isolates from blood cultures of 1201 children with suspected septicaemia in Calabar Nigeria

**Types of bacterial isolates**	Number of children with positive bacterial cultures in age groups (N = 552)					
	
	≤ 1 mo.	2 mo. – 1 yr	2 yr – 5 yr	6 yr – 10 yr	11 – 18 yr	Sub-total (% N)
*S. aureus*	138	52	51	22	6	269 (48.7)
*Coliforms*	63	24	33	7	2	129 (23.4)
Unidentified Gram-negative rods.	18	11	10	3	2	44 (8.0)
*Pseudomonas spp.*	16	5	9	2	0	32 (5.8)
β-haemolytic *Streptococci*	5	3	2	1	1	12 (2.2)
Other *Streptococci*	8	4	2	0	0	14 (2.5)
*Chromobacterium spp.*	15	5	2	2	1	25 (4.5)
*Salmonella typhi*	0	2	0	0	2	4 (0.7)
Other *Salmonella spp.*	5	3	0	1	0	9 (1.6)
CNS*	1	4	2	0	2	9 (1.6)
*Proteus spp.*	1	1	2	1	0	5 (0.9)
**Sub-total (% N)**	**271 (49.1)**	**113 (20.5)**	**113 (20.5)**	**39 (7.1)**	**16 (2.9)**	**552 (100)**

*CNS: Coagulase-negative *staphylococci*

Table 3 shows the anti-microbial sensitivity pattern of *Staphylococcus aureus *and *Coliforms *that made up 72% of all isolates. Antibiotic sensitivity data for the other isolates are not presented because they are too few. *Staphylococcus aureus *had the highest susceptibility to Ceftriazone (100%), Cefuroxime (100%), Azithromycin (100%), Erythromycin (90.1%) and Gentamicin (86.6%). *Coliforms *were most susceptible to Ceftazidime (78.8%), Ceftriaxone (83.3%), Cefuroxime (76.5%) and Azithromycin (92.9%).

**Table 3:** Antimicrobial sensitivity pattern of *Staphylococcus aureus *and *Coliforms*, the commonest bacterial isolates of children with septicaemia in Calabar Nigeria

Types of organism	% of isolates susceptible to antimicrobial agents*								
	
	A	C	E	Se	Ge	CFT	CFZ	CEX	Z
***Staphylococcus aureus***	4.4	57.6	90.1	28.5	86.6	66.9	100	100	100
	(136)	(159)	(182)	(123)	(179)	(115)	(30)	(65)	(19)
***Coliforms***	11.4	34.1	-	23.6	61.6	78.8	83.3	76.5	92.9
	(44)	(91)	-	(72)	(86)	(66)	(18)	(34)	(14)

*Figures in parenthesis show number of isolates tested.

Legend:

A = Ampicillin C = Chloramphenicol

E = Erythromycin Se = Cotrimoxazole

Ge = Gentamicin CFZ = Ceftriazone

CFT = Ceftazidine CEX = Cefuroxime

Z = Azithromycin

## Discussion

The present study includes children of all age groups but neonates are in the majority (44.4%). It reveals a rather high rate (44.9%) of isolation of bacterial pathogens from blood cultures of children with provisional diagnosis of septicaemia. The isolation rate is comparable to rates reported in other studies of Nigerian neonates with suspected septicaemia in Calabar (50.6%) [10], Ilorin (30.8%) [11] and Ife (55%) [12].

In all these reports, the organisms were *S. aureus *and gram-negative rods (*Pseudomonas aeruginosa *and *Escherichia coli*). The present study shows a similar pattern. This observation is in consonance with reports from two other developing countries [6,13] This suggests that infections by these agents constitute a significant threat to child survival in this locale and other developing country settings.

The *in vitro *susceptibility tests of these two most common isolates (*coliforms *and *S. aureus*) showed high levels of resistance to such commonly used antibiotics as Ampicillin, Chloramphenicol and Co-trimoxazole. While 86.6% of *S. aureus *isolates were sensitive to Gentamicin, only 61.6% of *coliforms *were sensitive to the same antibiotic, showing higher levels of resistance than reported in the same hospital about a decade ago, which showed a sensitivity of 89.7% [10,14]. The local antibiotics policy informed by the result of that study recommends the use of gentamicin as the sole agent for initial therapy in neonatal septicaemia. This policy needs to be reviewed to include a third generation cephalosporin in keeping with the result of the present study.

Susceptibility of *S. aureus *to Erythromycin remains high in the current study (90.1%). Sensitivity of the few isolates of *S. aureus *tested against Azithromycin is 100%, comparable to the findings of another Nigerian study [11]. This highlights the variable nature of antibiotic susceptibility pattern both in time and location within the same country.

Susceptibility of *coliforms *and *S. aureus *to third generation Cephalosporins (namely Ceftazidine, Cefuroxime and Ceftriazone), and Azithromycin were quite good. A recent report from Pakistan, another developing country showed high levels of resistance of gram-negative organisms to ceftazidime (71.6%) and cefotaxime (55.2%) [13]. Improved commitment to rational use of these antibiotics in Calabar is needed to sustain this relatively high level of susceptibility.

## Conclusion

This study has shown that *S. aureus *and gram-negative rods (*Pseudomonas spp *and *coliform*) are the leading causes of septicaemia in children in South-east Nigeria, a pattern similar to that of other low income countries. Observed decline in susceptibility of these common pathogens to common antibiotics calls for increased efforts to ensure more rational use of these drugs. Epidemiological surveillance studies such as the current one should provide useful information base to guide practice and policies on rational use of anti-infective agents.

## Competing interests

The author(s) declare that they have no competing interests.

## Authors' contributions

MMM, CEN and JUO participated in the clinical assessment and treatment of the patients. AEA and SJU supervised the laboratory procedures. CEN and MMM analysed the data. All the authors participated in the preparation of the final manuscript.

## Pre-publication history

The pre-publication history for this paper can be accessed here:


